# Effect of Additional Aluminum Filtration on the Image Quality in Cone Beam Computed Tomographic Studies of Equine Distal Limbs Using Visual Grading Characteristics Analysis: A Pilot Study

**DOI:** 10.3390/vetsci12111051

**Published:** 2025-11-02

**Authors:** Luca Papini, Mathieu de Preux, Frederik Pauwels, Joris Missotten, Elke Van der Vekens

**Affiliations:** 1 Swiss Institute of Equine Medicine (ISME), Department of Clinical Veterinary Science, Vetsuisse Faculty, University of Bern, 3012 Bern, Switzerland; lucapapini@icloud.com (L.P.); mathieu.depreux@unibe.ch (M.d.P.); 2 Plexus Veterinary Imaging, 39A Lloyd George Road Wainui, Gisborne 4010, New Zealand; fred@plexusveterinaryimaging.com; 3 Institute of Virology and Immunology, Department of Infectious Diseases and Pathobiology, Vetsuisse Faculty, University of Bern, 3012 Bern, Switzerland; joris.missotten@unibe.ch; 4 Institute of Virology and Immunology, 3147 Mittelhäusern, Switzerland; 5 Division of Clinical Radiology, Department of Clinical Veterinary Science, Vetsuisse Faculty, University of Bern, 3012 Bern, Switzerland

**Keywords:** fissure, horse, filter, diagnostic image quality assurance, tube current

## Abstract

(1) Background: Cone beam computed tomography (CBCT) is increasingly used in equine practice to diagnose musculoskeletal injuries, including fractures in the distal limb. However, limited detail in the thick cortical bone of the metacarpus/metatarsus hinders accurate diagnosis. In human medicine, the addition of aluminum filters (AF) enhanced image quality while reducing radiation exposure. This study aimed to evaluate the effect of AF on image quality in CBCT scans of equine distal limbs. (2) Methods: Adult equine cadaver limbs were scanned with a mobile CBCT unit using varying tube currents (10–100 mA) and AF (13–25 mm). Two independent experts assessed the image quality using a four-point visual grading scale, focusing on cortical bone detail and artifacts. (3) Results: Higher tube currents generally improved image quality, but no filter was beneficial for the metacarpal/metatarsal regions. For the proximal phalanx, thicker AF (19–25 mm) improved image quality without significantly increasing the required tube current. (4) Conclusions: The optimal balance between image quality and radiation exposure using the O-arm^®^ CBCT system for equine distal limbs was a tube current of 50 or 64 mA without filtration for the metacarpus/metatarsus, while a tube current of 50 mA with a 19–25 mm AF provided the best image quality for the proximal phalanx.

## Simple Summary

This pilot study aimed to improve the image quality in cone beam computed tomography (CBCT) for the diagnosis of fractures in equine distal limbs. Specifically, it evaluated the effects of varying tube current and aluminum filter thickness on image quality, including reductions in artifacts. Equine cadaver limbs were scanned using a mobile CBCT unit (O-arm^®^), with tube currents ranging from 10 to 100 mA and aluminum filters of 13 and 25 mm thickness. A board-certified veterinary radiologist and a board-certified equine surgeon, both blinded to the applied exposure and filters, evaluated the reconstructed images, focusing on the distinctness of anatomical structures and the presence of imaging artifacts. The results showed that higher tube currents generally improved image quality by reducing noise and enhancing the visibility of cortical and trabecular bone structures. However, overexposure was present at the periphery of the cortices at the highest tube current. For the metacarpal and metatarsal bones, the highest image quality was achieved without filters, particularly at tube currents of 50 or 64 mA. In these regions, the application of filters actually reduced image quality, likely due to diminished X-ray penetration through thick cortical bone. In contrast, for the proximal phalanx (P1), the use of thicker aluminum filters (19–25 mm) at 50 mA enhanced image quality and reduced artifacts. These findings provide preliminary recommendations for optimizing CBCT protocols in equine distal limb imaging using the O-arm^®^. Moderate tube currents (50–64 mA) without filters were ideal for scanning the metacarpal and metatarsal bones, while thick aluminum filters used at 50 mA yielded superior results for P1. These suggested settings balance diagnostic quality with radiation safety, representing a first step toward more accurate detection of subtle bone lesions and fractures in long bones in the distal limb, and aim to contribute to earlier diagnosis and improved clinical outcomes in equine orthopedic practice.

## 1. Introduction

Computed tomography (CT) and cone beam CT (CBCT) are cross-sectional diagnostic imaging techniques that have been increasingly used in clinical equine practice in recent years [1,2]. Their application has been described for the identification of pathological changes in the equine skull, specifically lesions affecting osseous structures, teeth, nerves, and peripheral soft tissues, and for lameness work-ups revealing musculoskeletal injuries [3,4,5,6,7]. The higher contrast resolution of CT images compared to radiographic images, and the elimination of superimposition, provide CT with significant diagnostic value in the diagnosis of fractures and fissures occurring at the level of the equine distal limbs and the equine head [8]. However, at the Vetsuisse Faculty of the University of Bern, it was observed that osseous fissures can go undiagnosed in the acute stage on CBCT images acquired with the O-arm^®^ imaging unit (Medtronic, Louisville, CO, USA). This is due to blooming and/or insufficient spatial resolution in the thick cortex of long bones, specifically in the metacarpus/metatarsus, which can have a detrimental effect on the diagnosis and treatment of clinical cases. Pre-filtering of the X-ray beam as a tool to improve image quality and decrease patient dose has been mainly studied in humans, specifically in pediatric medicine [9,10,11]. This process, known as “beam hardening”, uses filters to absorb low-energy X-rays, which reduces beam hardening artifacts and patient dose. The thickness and material of the filter influence the degree of filtration and the effect on image quality; primarily, aluminum and copper filters have been evaluated [9,12,13]. A possible disadvantage of excessive filtration is an increase in statistical noise due to the reduced number of photons available for image formation and the associated decrease in image quality [9]. Previous studies have evaluated the effect of exposure values on the image quality of equine head CT [14]. However, to the best of the authors’ knowledge, the potential benefits of added filtration and different tube currents have not yet been evaluated in CBCT of equine distal limbs.

The purpose of this study was to determine the optimal exposure parameters and filter thickness for CBCT images acquired with the O-arm^®^ imaging unit of the Vetsuisse Faculty of the University of Bern (O-arm^®^; Medtronic) in order to improve cortical bone detail in equine distal limbs. 

## 2. Materials and Methods

### 2.1. Image Acquisition and Reconstruction

**Figure 1:**
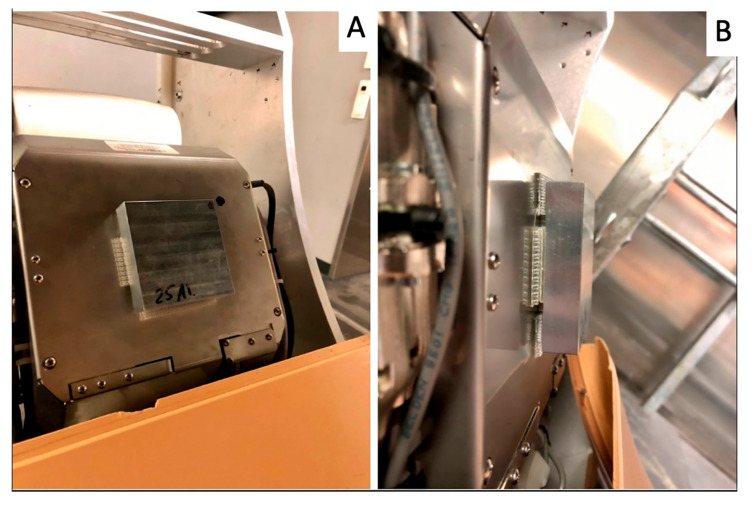
A 25 mm thick aluminum filter installed on the X-ray tube housing using a heavy-duty reclosable fastener; front (**A**) and side (**B**) view.

Adult equine cadaver limbs (*n* = 3), donated by the owners with a signed consent for research purposes and euthanized at the Swiss Institute of Equine Medicine (ISME), University of Bern, for reasons unrelated to this study, were used. Four anatomical regions were scanned for this study: 2 metacarpi (MC and MC-S), 1 metatarsus (MT), and 1 proximal phalanx (P1). The same distal forelimb was used for the MC and P1 regions, and also the MT originated from the same warmblood horse, while MC-S originated from a Shetland pony. Each limb was positioned on a carbon fiber table (Opera Swing; General Medical Merate SPA, Seriate, Italy). Image acquisition was performed using a mobile CBCT unit (O-arm^®^; Medtronic). Orthogonal fluoroscopic views were obtained to center the chosen region in the center of the field of view before starting the sequence of scans. Each anatomical region was subsequently scanned using the Standard 3D-knee mode, with a fixed power setting of 125 kV and manually varied tube currents (11 settings: 10, 12, 16, 20, 25, 32, 40, 50, 64, 80, and 100 mA). After the native scanning sequence, a mushroom-shaped aluminum filter consisting of a large square (7.7 × 7.7 cm) and a smaller square (4.7 × 4.7 cm), produced by RECO Mecanique SA, Salgesch, Switzerland, was installed on the tube housing. For fixation, a velcro-type reclosable fastener (3M SJ-3560 Dual Lock, Industrial Adhesives and Tapes Division, 3M Center, Maplewood, MN, USA) was applied on both the larger square of the filter and the tube housing, as shown in Figure 1A,B.

**Figure 2:**
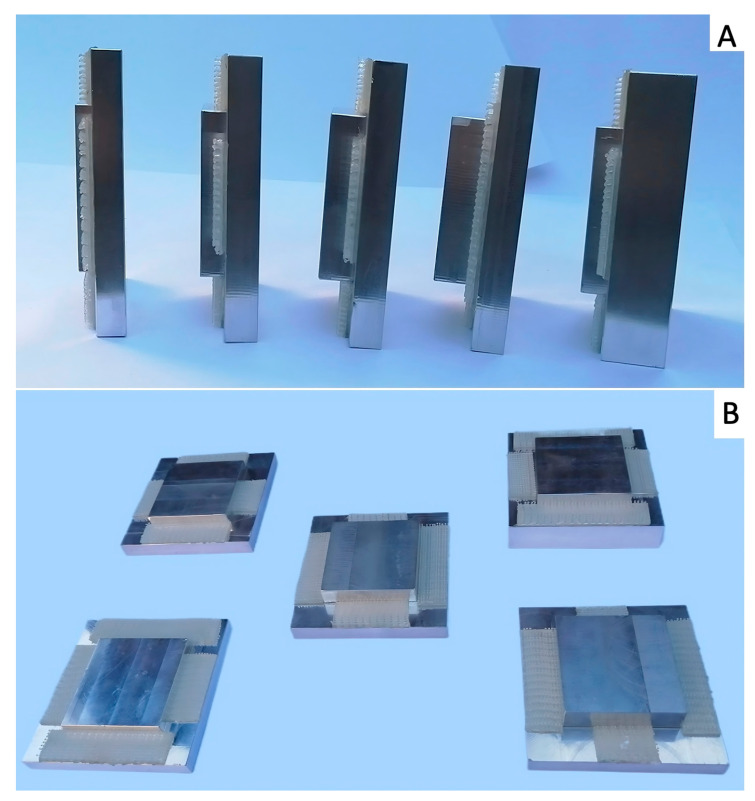
(**A**) Side view of the five mushroom-shaped aluminum filters, increasing thickness from left to right (13 mm, 16 mm, 19 mm, 22 mm, and 25 mm). (**B**) Front view of the same filters as in (**B**), showing the reclosable fastener used to fix the filters to the tube housing.

For the latter, the O-arm^®^ gantry was opened, and the tube housing was made accessible using the available lever arm, as performed during technical maintenance. The smaller square filter fits inside the tube housing, allowing thicker filters to be used without risking contact between the filter and the inside of the CT gantry during tube rotation. A total of 5 filters with different thicknesses (13 mm (F13), 16 mm (F16), 19 mm (F19), 22 mm (F22), and 25 mm (F25)) were applied consecutively (Figure 2A,B); the same sequence of scans was repeated for each filter using the parameters listed above. 

Therefore, a total of 264 image series were created (4 bones, 1 native, 5 filters, and 11 current settings). Each series consisted of 192 reconstructed isometric transverse CT images numbered iNr 1 to 192, based on 391 exposures made during a 13 s acquisition period and a 360° tube rotation. These reconstructed images had a slice thickness of 0.833 mm and an in-plane resolution of 0.415 mm.

All computed tomographic image series were exported and stored in a picture archiving and communication system (PACS, DeepUnity Diagnost, Dedalus Healthcare Group) and evaluated on dedicated imaging workstations in DICOM format. 

### 2.2. Data Recording and Analysis

To allow blinded image evaluation by external observers, a postgraduate equine clinician (LP) selected, in consensus with a non-blinded board-certified veterinary radiologist (EVdV), the three image series with different tube currents that provided the best subjective image quality. This selection was made for each of the 4 anatomical regions and for both the native scans and all filter thicknesses. The selected series were anonymized and subsequently assigned a random identification number (*n* = 72; 18 series per bone, including 3 series per filter and 3 series for the ‘native’ group). Fixed window width (WW) and window level (WL) settings (WL: 2500; WW: 6000), subjectively considered appropriate during preliminary evaluation and selection of the scans by the non-blinded board-certified veterinary radiologist, were used during all further evaluations.

#### 2.2.1. Visual Image Quality Assessment 

Blinded, independent quality assessments of the 72 reconstructed series were performed by one board-certified veterinary radiologist (FP) and one board-certified equine surgeon (MdP). For each series, either 3 areas for the metacarpi/-tarsi (MC/MC-S/MT) and 2 areas for P1 of 10 consecutive slices each, were provided for evaluation: the proximal meta-/diaphysis (iNr. 32–42), mid diaphysis (iNr. 91–101) and distal meta-/diaphysis (iNr. 150–160) of MC/MC-S/MT, respectively, proximal meta-/diaphysis and mid to distal diaphysis of P1 (iNr. 88–98 and 115–125). Image quality was assessed according to a four-point visual grade scale shown in Table 1.

**Figure 3:**
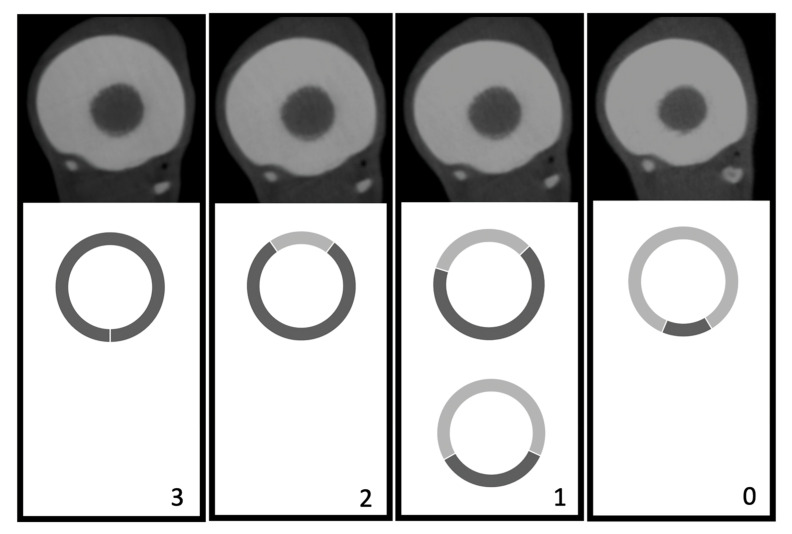
Distinctness of anatomical structures: (3) complete internal structure (IS), 0% loss of IS; (2) nearly intact IS, 1–25% loss of IS; (1) 25–75% loss of IS; and (0) >75% loss of IS.

This grading system was adapted from the system described by Demehri et al. (2015) [15]. Specifically, the visibility of trabecular pattern within the cortex of long bones (distinctness of anatomical structures) was graded on a four-point scale as a sign of sufficient penetration of the X-ray beam: (3) complete internal structure (IS), 0% loss of IS; (2) nearly intact IS, 1–25% loss of IS; (1) 25–75% loss of IS; and (0) >75% loss of IS (Figure 3).

**Figure 4:**
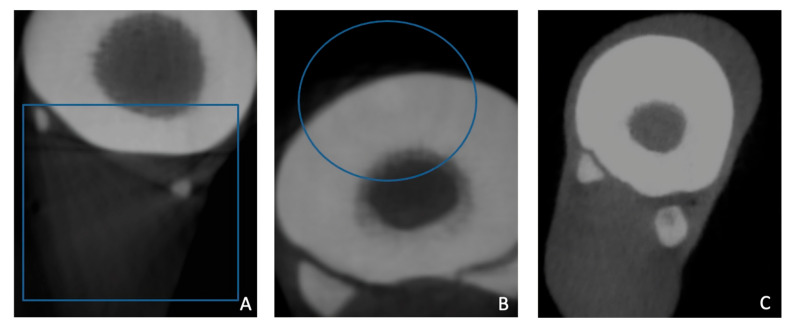
Different artifacts were observed in the CBCT scans, including streak artifacts (blue square) (**A**), cone beam artifacts (blue circle) (**B**), and quantum noise (**C**).

Each observer recorded their scores in a predesigned spreadsheet (Microsoft Excel; Supplementary Material). In addition, the observers had to indicate whether any artifacts were visible that hampered cortical evaluation; these included significant quantum noise, cone beam artifacts, streak artifacts, or other artifacts (Figure 4).

**Figure 5:**
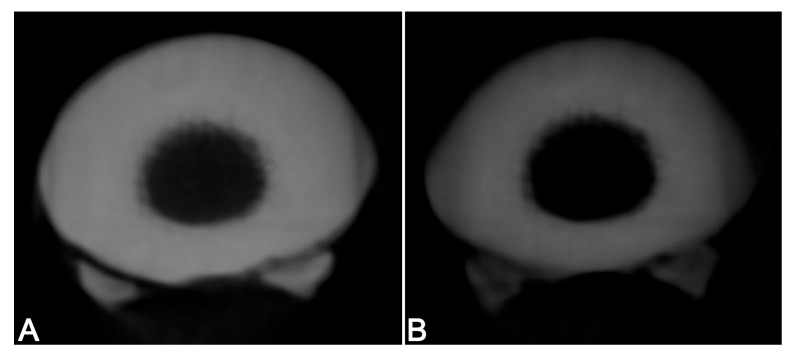
Native cone beam computed tomographic scans of MC using a fixed tube potential of 125 kV and either a 64 mA (**A**) or a 100 mA (**B**) tube current; a good distinction of internal structure (grade 4) is present in the cortex of images A and B, but image B is overexposed, with clear edge burn-off at the bone margins. Image B, which would be graded as a 1 for artifacts, was not selected as a tube current to be scored by the blinded observers.

Also, the overexposure, as seen in Figure 5, needed to be noted, as it could affect the visibility of the outer cortex and thereby hinder the accurate diagnosis of a possible fissure and potentially associated early periosteal reaction. 

The presence of artifacts was graded on a 4-point scale: (3) no artifact; (2) slight artifact, with minimal effect on IS recognition; (1) moderate artifact, slightly impaired recognition of IS; and (0) prominent artifact, severely impaired recognition of the IS. 

After a first individual assessment, the two board-certified experts re-evaluated the areas where their scores differed by more than 1 grade. For these areas, a second assessment was performed by both experts together to reach a consensus. 

#### 2.2.2. Clinical Case

The right metatarsus (MtF) of a 12-year-old Dutch warmblood horse, euthanized following the radiographic diagnosis of a metatarsal fissure and rejection by the owners to opt for surgical treatment, was used. Image acquisition was performed using the same scan protocol as for the first part of the study, including the 11 different current settings to acquire a native series, and repeated scans applying the same 5 filters. The scans were then evaluated by the postgraduate equine clinician (LP) together with the non-blinded board-certified veterinary radiologist (EVdV) to identify the series with the best image quality and clearest visibility of the fissure using the same software and settings. 

#### 2.2.3. Statistics 

The interobserver variation in the visual image quality assessment of both observers was compared using a paired *t*-test. The best performing filter and tube current were determined using a Bayesian ordinal regression model (JASP software, Version 0.19.3.0 for Windows) based on the visual grade analysis scores of both observers, with the score as the ordinal factor. This model was applied for the MC, MC-S, and MT data with either the filter or the tube current as a fixed factor. Both calculated filter and current effects were then combined, assuming additive contributions from both predictors, into a performance matrix.

## 3. Results

### 3.1. Visual Image Quality Assessment

Both blinded reviewers assessed all 72 reconstructed CBCT scans. In cases where their initial scores differed by more than one grade, a joint re-evaluation was conducted to reach consensus. However, even after this targeted reassessment, statistically significant differences remained between the observers’ scores in both evaluation categories—image quality and artifact presence (*t*-test, *p* < 0.05). The percentage of agreement, calculated as the proportion of identical ratings out of the total, was 61% for “Distinctness of anatomical structures” and 70% for “CT artifacts”. As a result, average scores were not computed; individual scores were retained for subsequent evaluations and statistical analysis. 

The results of the comparisons between the combinations of the different filters, including no filter and the different tube currents for the MC, MT, and MC-S, are shown in the performance matrix in Table 2.

The native scan series clearly outperformed all filters, with results similar for tube currents of 64 and 50 mA. There was a slight preference for 50 mA, as despite a lower visibility score for IS, little to no peripheral overexposure was observed. When only IS grades were taken into account, the native scans with a tube current of 64 mA scored better than those with 50 mA, with average scores of 2.66 versus 2 on a maximum grade of 3, respectively. Also, both F16 and F13, when combined with 50 mA of tube current, performed better than average. Filter thicknesses from 13 mm to 22 mm in combination with 64 mA, as well as filters F19 and F22 in combination with 50 mA, performed around average. The tube current setting of 100 mA, combined with a 25 mm aluminum filter, performed worst, followed by F19 and F22.

For P1, tube currents 40, 50, and 64 were evaluated for the native and all filters except F13, where 50, 64, and 80 were selected. None of the scans with filters showed artifacts that influenced the evaluation of the internal structure, and all were graded the maximum score of 3 by both observers. However, the native scans at both 50 and 64 mA showed peripheral overexposure, and 1 observer noted streak artifacts. Therefore, both 50 mA and 64 mA scored worse with average artifact scores of 1.5 and 0.5 out of a maximum of 3, compared to 40 mA, which attained an average artifact score of 2.5. All filters from 19 mm thickness upwards had a perfect score for both artifacts and internal structure at 50 mA, while either the lower (for F22) or higher current (for F19) was scored slightly worse for IS by at least one of the observers, creating a mean overall score of 2.75 and 2.5, respectively. For filter thicknesses of 16 mm or less, including the native scans, none of the evaluated tube currents produced images that received a perfect score for internal structure from either observer.

Applying a filter reduced artifacts across all four anatomical regions, primarily preventing peripheral overexposure.

### 3.2. Clinical Case–Visual Image Quality Assessment

**Figure 6:**
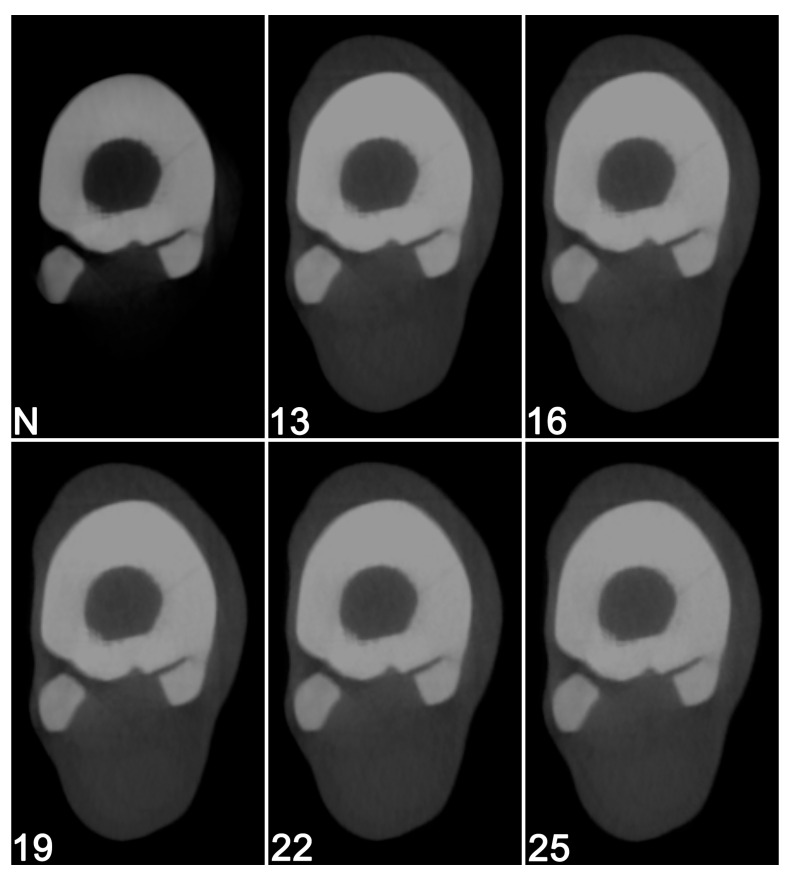
Cone beam computed tomographic scans of a right metatarsal bone with a naturally occurring acute fissure using a fixed tube potential and current of 125 kV and 64 mA, but with aluminum filters of different thicknesses. N = No filter (native scan); 13 = 13 mm; 16 = 16 mm; 19 = 19 mm; 22 = 22 mm; 25 = 25 mm filter. The native scan without filter was considered to provide the best visibility of the fissure and the internal structure within the adjacent dorsal cortex.

**Figure 7:**
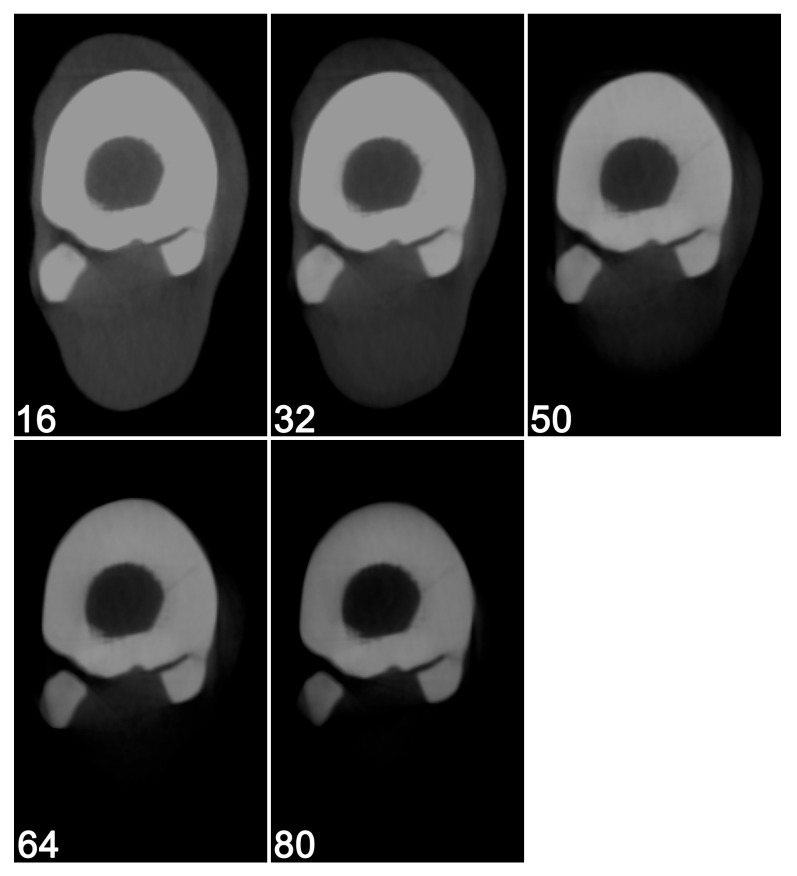
Five native cone beam computed tomographic scans of a right metatarsal bone with a naturally occurring acute fissure using a fixed tube potential of 125 kV, but different current settings:16 mA, 32 mA, 50 mA, 64 mA, and 80 mA, respectively. A tube current of 64 mA was considered the lowest exposure that provided a good visibility of the fissure as well as the internal structure of the adjacent dorsal cortex.

When comparing the different scans obtained in MtF using the different filters and current settings, the results from the first part of the study could be confirmed. The fissure was indeed best visible in the native scans (Figure 6) and on the scans with the highest mA (Figure 7). 

## 4. Discussion

This study focused on identifying CBCT acquisition parameters that enhance the detection of subtle fissures by using variable tube currents and additional filtration, specifically in challenging anatomical regions characterized by dense, thick cortical bone, such as the third metacarpal/-tarsal bones and the proximal phalanx of equine limbs. Modifying only the tube current directly influences the image noise and contrast without altering other acquisition parameters that could confound the interpretation of results. By isolating this variable, we were able to specifically assess its effect on fissure detection, ensuring that any observed differences in image quality could be attributed solely to changes in tube current. In our standard protocol, the tube power (125 kV) was fixed and could not be modified. Alternative scan modes, such as high-resolution, are possible on the O-arm^®^ and would allow different power settings but also require substantially longer scan times, making them unsuitable for scanning standing sedated horses.

Higher tube currents were generally associated with improved image quality across all evaluated regions. This supports the need for high tube currents to avoid missing fine fissures. However, higher currents entail increased radiation exposure for animal handlers in the room, who must position the limb correctly during scanning of standing, sedated horses [6]. 

While native (unfiltered) scans provided the best image quality in the MC, MC-S, and MT regions, thicker aluminum filters (19 mm, 22 mm, or 25 mm) enhanced image quality in the P1 region without requiring increased exposures. The filters also had a positive effect by reducing artifacts, especially overexposure at the edges of the cortex in native scans, but the decreased penetration and associated reduced diagnostic quality of the thick cortical bone in the third metacarpal/-tarsal bones outweighed the benefits of reducing artifacts. As a compensatory means, achieving a high-quality image with filters required increasing the tube current beyond that used for native images. Therefore, the use of filters is not in line with the ALARA principle of radioprotection for personnel and helpers during scanning of equine metacarpi and -tarsi. 

Based on the results of our study, we propose practical guidelines for CBCT imaging of equine limbs acquired using the O-arm^®^ imaging unit, as outlined in Table 3. 

A key component of this study was the use of a custom visual grading scale for image quality analysis. Visual Grading Analysis (VGA) is a robust, observer-based tool frequently used in radiological research to evaluate the clinical adequacy of images, particularly in relation to dose optimization strategies [15,16]. In our study, the grading scale was tailored to focus on anatomical detail relevant to equine limb pathology, with particular emphasis on trabecular pattern visibility and artifact interference.

The interobserver agreement of this study was fair to moderate, preventing the combination of their results. This result is lower than a previous study comparing CBCT and multidetector CT for the visualization of anatomical structures in the fetlock region [17]. However, that study focused on the visibility of a structure—its margins, shape, size, and overall attenuation—without specifically evaluating the internal structure of the cortices. Our results mirror findings in human CBCT imaging, where interobserver agreement levels below the commonly accepted threshold of 80% have also been reported for visual assessments of bone and soft tissue structures [15]. These results underline the subjective nature of visual image quality assessment and the importance of using combined quantitative and qualitative evaluation approaches or of additional validation, especially when introducing technical modifications like filtration. While the gray scale values of the O-arm^®^ CBCT system do not represent Hounsfield units (HUs), quantitative measurements may still be applied when comparing regions of diseased versus non-diseased bones [18]. However, that approach cannot be used in this study when evaluating the effects of imaging parameters and filters on the same bone. We used post-mortem CBCT results from a clinical case with an in vivo-diagnosed fissure (MtF) to provide additional validation for part of our findings from the visual image quality assessment study. The metatarsal bone fissure was optimally visualized with a tube current of 64 mA without additional filtration.

This study has several limitations that should be acknowledged. Although both the number of limbs and evaluators are the same as a previous study comparing the diagnostic quality of CBCT and fan-beam CT in the equine metacarpophalangeal joint [19], and the number of reviewers is similar to other studies evaluating image quality in equine CT examinations [20,21], the sample size was small. We evaluated only three cadaver limbs and one clinical case, which limits the extrapolation of the findings to the broader equine population. We specifically included a pony limb as one of the normal cadaver limbs, but no limbs of draft horses were included. Although previous studies have shown differences in density between horse breeds in the navicular and carpal bones, a recent study did not detect such differences in the metacarpus, which is in line with our observations [22,23,24]. However, evaluating a larger number of limbs from different breeds would allow further confirmation of these results. Similarly, only two blinded evaluators performed the analysis of visual grading characteristics. Despite these low numbers, clear trends toward higher required tube currents and no added filtration were observed in the metacarpus and -tarsus regions in this preliminary study. However, future studies should include a larger number of equine limbs and a greater number of blinded evaluators to strengthen the reliability of the results. Another limitation is the lack of quantitative image quality metrics, such as signal-to-noise ratio (SNR) and contrast-to-noise ratio (CNR). Previous studies have used the mean gray scale voxel value and its standard deviation to calculate CNR [18,25]. In addition, scanning phantoms can be used to calculate the SNR. Performing these evaluations could provide additional quantitative information on the effect of filtration on CBCT image quality. Finally, these results are specific to the O-arm^®^ system and cannot be extrapolated to other CBCT systems. Specifically, post-processing varies across CBCT systems, as the selected protocol and associated reconstruction method significantly influence the appearance of the generated transverse CBCT images. In the authors’ experience with the O-arm 2® system, these post-processing algorithms can play a major role in the conspicuity of internal structures within the cortex, and they cannot be altered retrospectively. Therefore, future work should explore the use of different post-processing algorithms, including artificial intelligence-based image processing techniques, which have been shown to improve image quality and diagnostic value in other fields [26]. Radiation dose optimization studies would also be valuable to ensure the safe and effective clinical use of CBCT in equine practice.

## Figures and Tables

**Table 1:** Image quality assessment using a visual image characteristics analysis with 4 grades. IS: Internal structure. CT artifacts: noise artifact, cone beam artifact, streak artifact, overexposure, and other artifacts.

Variable	Rating
Distinctness of anatomical structure	3. Complete IS
	2. Near all IS, <25% loss of IS
	1. 25–75% loss of IS
	0. >75% loss of IS
CT artifacts	3. No artifact
	2. Slight artifact, with minimal effect on IS recognition
	1. Moderate artifact, slightly impaired recognition of IS
	0. Prominent artifact, severely impaired recognition of the IS

**Table 2:** Combined filter x mA current performance matrix for the MC, MT, and MC-S, taking both the scores for distinctness of anatomical structure (IS) and CT artifacts into account. N = Native scan; F13 to 25 represent the added aluminum filters with thicknesses varying from 13 to 25 mm.

	mA	50 mA	64 mA	80 mA	100 mA
Filter					
N		0.610	0.601	0.321	0.048
F13		0.177	0.168	−0.112	−0.385
F16		0.188	0.179	−0.101	−0.374
F19		0.078	0.069	−0.211	−0.484
F22		0.179	0.170	−0.110	−0.383
F25		0.003	−0.006	−0.286	−0.559

**Table 3:** Guidelines for CBCT imaging of equine limbs with the O-arm^®^ imaging unit.

Region	Recommended Tube Current	Filter
Metacarpus/Metatarsus	50–64 mA	None (native scan)
Proximal Phalanx (P1)	50 mA	Aluminum filter (19–25 mm)

## Data Availability

The original contributions presented in this study are included in the article/Supplementary Materials. Further inquiries can be directed to the corresponding author.

## Supplementary Materials

The predesigned spreadsheet used for scrorring of all the scans has been submitted as supplementary material together with the manuscript to MDPI and is available for consultation. The following supporting information can be downloaded at: https://www.mdpi.com/article/10.3390/vetsci12111051/s1.

## Abbreviations

The following abbreviations are used in this manuscript:

CBCT	Cone beam computed tomography
IS	Internal structure
MT	Metatarsus
MC	Metacarpus
MC-S	Metacarpus small
P1	Proximal phalanx
