# Supplementary material for: Effect of Additional Aluminum Filtration on the Image Quality in Cone Beam Computed Tomographic Studies of Equine Distal Limbs Using Visual Grading Characteristics Analysis: A Pilot Study
Source: Vet Sci. 2025 Nov 2;12(11):1051. doi: 10.3390/vetsci12111051 (PMC12656789; doi:10.3390/vetsci12111051)
Supplement: Supplementary file 1 [file vetsci-12-01051-s001.zip › vetsci-3849452-supplementary.pdf]

Patientenname	BildNr. 32-42	BildNr. 91-101	BildNr. 150-160	BildNr. 32-42	BildNr. 91-101	BildNr. 150-160	significant noise artefact ?	cone beam artefact ?	streak artefacts ?	other artefacts?	Comments
f1											
f2											
f3											
f4											
f5											
f6											
f7											
f8											
f9											
f10											
f11											
f12											
f13											
f14											
f15											
f16											
f17											
f18											

Patientenname	BildNr. 32-42	BildNr. 91-101	BildNr. 150-160	BildNr. 32-42	BildNr. 91-101	BildNr. 150-160	significant noise artefact ?	cone beam artefact ?	streak artefacts ?	other artefacts?	Comments
t1											
t2											
t3											
t4											
t5											
t6											
t7											
t8											
t9											
t10											
t11											
t12											
t13											
t14											
t15											
t16											
t17											
t18											

Patientenname	BildNr. 32-42	BildNr. 91-101	BildNr. 150-160	BildNr. 32-42	BildNr. 91-101	BildNr. 150-160	significant noise artefact ?	cone beam artefact ?	streak artefacts ?	other artefacts?	Comments
s1											
s2											
s3											
s4											
s5											
s6											
s7											
s8											
s9											
s10											
s11											
s12											
s13											
s14											
s15											
s16											
s17											
s18											

Patientenname	BildNr. 88-98	BildNr. 115-125		BildNr. 88-98	BildNr. 115-125		significant noise artefact ?	cone beam artefact ?	streak artefacts ?	other artefacts?	Comments
p1											
p2											
p3											
p4											
p5											
p6											
p7											
p8											
p9											
p10											
p11											
p12											
p13											
p14											
p15											
p16											
p17											
p18											

Variable	Rating
Distinctness of anatomical structures	3. Perfect internal structure
	2. Near all IS, < 25% loss of internal structure
	1. 25 – 75 % loss of internal structure
	0. > 75 % loss of internal structure

Variable	Rating
CT artefacts influencing the cortical evaluation	3. No artefact
	2. Slight artefact, with minimal effect on parenchymal recognition
	1. Moderate artefact, slightly impaired recognition of parenchyma
	0. Prominent artefact, severely impaired recognition of the parenchyma
